# Unraveling the relationship between the renin–angiotensin system and endometrial cancer: a comprehensive review

**DOI:** 10.3389/fonc.2023.1235418

**Published:** 2023-10-05

**Authors:** Nihad Ashraf Khan, Deena Elsori, Gowhar Rashid, Sonia Tamanna, Ananya Chakraborty, Adeeba Farooqi, Ayman Kar, Niti Sambyal, Mohammad Azhar Kamal

**Affiliations:** ^1^ Department of Biosciences, Faculty of Natural Sciences, Jamia Millia Islamia, Delhi, India; ^2^ Faculty of Resillience, Deans Office Rabdan Academy, Abu Dhabi, United Arab Emirates; ^3^ Amity Medical School, Amity University, Gurgaon, Haryana, India; ^4^ Department of Biochemistry and Molecular Biology, University of Dhaka, Dhaka, Bangladesh; ^5^ Department of Biotechnology, Adamas University, Kolkata, West Bengal, India; ^6^ Department of Biotechnology, Central University of Kashmir, Ganderbal, India; ^7^ Department of Biotechnology, Shri Mata Vashino Devi University, Katra, Jammu, India; ^8^ Department of Pharmaceutics, College of Pharmacy, Prince Sattam Bin Abdulaziz University, Alkharj, Saudi Arabia

**Keywords:** RAS pathway, angiotensin I-II, ACE, immunosuppressor, endometrial cancer

## Abstract

Endometrial cancer (EC), the most common adenocarcinoma, represents 90% of uterine cancer in women with an increased incidence of occurrence attributed to age, obesity, hypertension, and hypoestrogenism. Being the most common gynecological malignancy in women, it shows a relation with the activation of different components of the renin–angiotensin system (RAS), which is predominantly involved in maintaining blood pressure, salt, water, and aldosterone secretion, thereby playing a significant role in the etiology of hypertension. The components of the RAS, i.e., ACE-I, ACE-II, AT1R, AT2R, and Pro(renin) receptor, are widely expressed in both glandular and stromal cells of the endometrium, with varying levels throughout the different phases of the menstrual cycle. This causes the endometrial RAS to implicate angiogenesis, neovascularization, and cell proliferation. Thus, dysfunctioning of the endometrial RAS could predispose the growth and spread of EC. Interestingly, the increased expression of AngII, AGTR1, and AGTR2 showed advancement in the stages and progression of EC via the prorenin/ATP6AP2 and AngII/AGTR1 pathway. Therefore, this review corresponds to unraveling the relationship between the progression and development of endometrial cancer with the dysfunction in the expression of various components associated with RAS in maintaining blood pressure.

## 1 Introduction

Endometrial cancer (EC), the most common female uterine cancer, mainly arises in post-menopausal women with an average diagnostic age of 60 years. According to the American Cancer Society, 2021 marked the occurrence of 66,570 new cases of EC in the US and more than 12,940 deaths. The increasing incidence of EC and its estimated growth rate in subsequent years have posed a great threat to the public health sector (1). It has been estimated that over 90% of uterine cancers are adenocarcinomas, of which ~80% are associated with an increased expression of estrogen under the influence of insulin resistance and obesity, whereas 20% are of unknown etiologies. The excess of exogenous and endogenous estrogens corresponds to the main risk factors for endometrial adenocarcinoma (2). Additionally, most of the past studies focused on evaluating the expression of estrogen and progesterone receptors (ER and PgR) in EC. These studies predominantly showed that 85%–90% of the well-differentiated ECs were positive for ER/PgR, whereas 70%–85% of moderately differentiated ECs expressed steroid receptors. Additionally, only 13% of poorly differentiated EC had detectable levels of ER/PgR, thereby assigning these receptors as predictive and prognostic biomarkers against anti-hormonal therapy in EC (3).

Among the various malignancies worldwide, EC has been rated as the seventh most common malignancy after breast, lung, and colorectal cancer. It differs into various histological subtypes based on varying frequency, clinical presentation, and prognosis as well as associated epidemiological risk factors (4). Endometrioid EC, serous EC, clear-cell EC, mixed EC, and uterine carcinosarcoma (UCS) correspond to different histological subtypes of EC. Endometriosis, significantly characterized by the presence of functionally active endometrial tissue, stroma, and glands outside the uterine cavity, is an estrogen-dependent inflammatory disorder of the endometrium affecting up to 11% of women of reproductive age worldwide, whereas its etiology remains largely unknown. However, among the several theories proposed to explain the pathogenesis of endometriosis, retrograde menstrual blood flow, coelomic metaplasia, and Mullerian remnants are famous (5). Apart from these theories, it has been reported that the components of the renin–angiotensin system (RAS), which are famously responsible for maintaining normal blood pressure and sodium homeostasis, are expressed in the endometrium during pregnancy. Moreover, emerging research suggests the potential implications of RAS in various gynecological cancers such as ovarian and breast cancer, indicating a broader role of RAS in influencing cellular processes critical to the progression of various gynecological malignancies. This RAS activity has been shown to stimulate angiogenesis, cell proliferation, and migration within the normal endometrium. Notably, dysfunction in the expression of these RAS components within the endometrium has also been associated with the development of endometrial cancer in women (6). A study conducted by Piastowska-Ciesielska et al. demonstrated that the levels of expression of AGTR1, AGTR2, vascular endothelial growth factor (VEGF), and estrogen receptor (ER)-α levels varied with the different stages of cancer, concluding a higher expression of AngII receptors in the early grade of EC (1). Additionally, Shibata et al. showed the levels of AngII, AGTR1, and VEGF peptides along with adipocyte-derived leucine aminopeptidase (A-LAP) as prognostic for EC (7). Significantly, (pro) renin receptor (PRR), a new biomarker for different types of cancer such as colorectal cancer, breast cancer, glioma, aldosterone-producing adenoma, urothelial cancer, and pancreatic ductal adenocarcinoma, has also been regarded as a potential prognostic and therapeutic biomarker for endometrial cancer (8). In various physiological and pathological pathways of tumorigenesis, (pro) renin receptor plays important roles via the Wnt/β-catenin, renin–angiotensin system, MAPK/ERK, and PI3K/AKT/mTOR pathways. Interestingly, our limited knowledge about PRR in cardiovascular and renal physiological functions and diseases has been updated with compelling pieces of evidence stating the prominent role of PRR in various cancers including endometrial cancer (9).

Moreover, renin–angiotensin–aldosterone system (RAAS) inhibitors, angiotensin-converting enzyme inhibitors (ACEIs), and antagonists of angiotensin receptors (ARBs) have been strongly associated with a considerable decline in the overall risk of gynecologic cancer. This system has a high potential to be expressed and/or triggered to promote aberrant cell growth and dissemination responsible for boosting angiogenesis, cell proliferation, and migration, a significant characteristic of endometrial cancer (10).

## 2 The renin–angiotensin system

**Figure 1:**
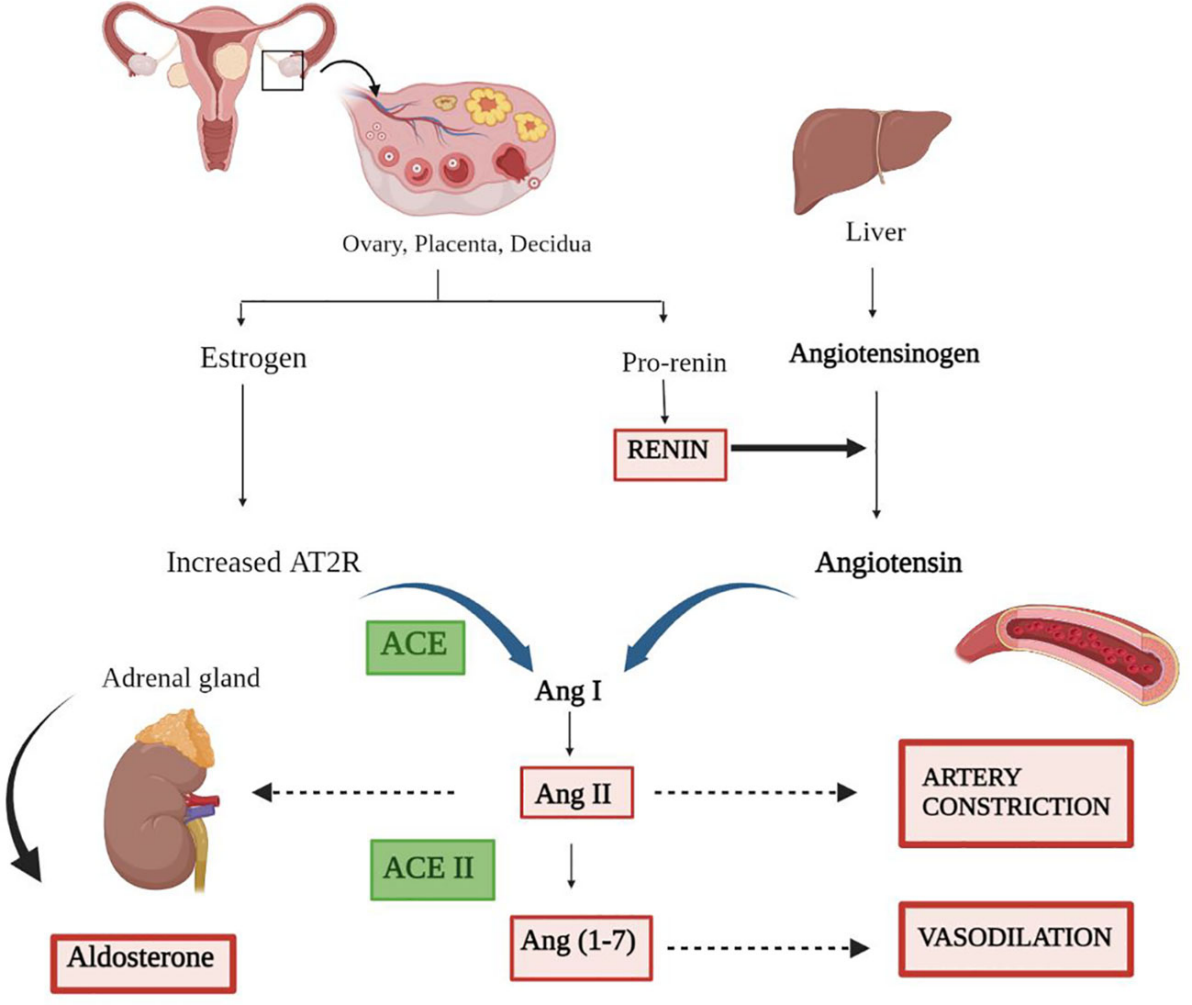
Representation of the renin–angiotensin system. The secretion of estrogen and pro-renin from the ovary, placenta, and decidua through a series of events results in artery constriction and vasodilation. Renin, the major candidate responsible for converting inactive angiotensinogen to active Angiotensin, is produced by the liver. Furthermore, the combined effect of angiotensin and receptor for angiotensin (AT2R) convert AngI to AngII via ACE. ACE II comes into play and catalyzes the final conversion of AngII to Ang (1–7), ending in a variety of responses along with the production of aldosterone.

The RAS is a complex and vital biochemical pathway that maintains plasma sodium concentration, arterial blood pressure, and extracellular volume. The RAS consists of three main components: angiotensinogen, renin, and angiotensin-converting enzyme (ACE) (**Figure 1**) (11). Renin, a hormone secreted by the kidneys, acts on its substrate to catalyze the transformation of angiotensinogen into angiotensin I. Angiotensin I is known to be a powerful vasoconstrictor that contributes to the development of hypertension. The conversion of angiotensin I to angiotensin II occurs under the influence of the ACE produced by the lungs. The RAAS is a group of genes and proteins that control the body’s fluid balance and blood pressure. The RAAS network is responsible for releasing renin into the bloodstream, thereby catalyzing the conversion of angiotensinogen to angiotensin I. RAAS activation greatly shows its effect like controlled blood pressure, cell proliferation, inflammation, and fibrosis on every organ. Thus, an imbalance of renin and angiotensin II can be a leading cause of several chronic and acute diseases (12).

The prorenin sensor is a prominent regulatory protein synthesized by the kidneys that helps to regulate blood pressure by maintaining blood flow in the body’s blood vessels and cells. It is also found in ovarian follicular fluid in extremely higher concentrations. Thus, the prorenin plasma levels increase transiently in blood during the menstrual cycle in the luteal phase. This provides a close relationship between ovarian prorenin levels in reproductive function, showing a deep relevance with female infertility, endometriosis, and toxemia during pregnancy. The gene REN in humans produces the protein known as the renin transmitter along with the renin receptor family protein (13).

Due to the presence of ovarian prorenin, the RAS is believed to play a role in the development of endometrial cancer when dysregulated (14). In the last few years, researchers have made great strides in understanding how cells communicate with each other and focused on understanding the response of cells to specific signals. One particularly important type of communication involves the crosstalk between the RAS pathway and the progression of endometrial cancer.

## 3 RAS and convergent signaling pathways in endometrial cancer

Different signaling pathways have an immense impact on endometrial cancer. The convergent pathways allow cells to share information more efficiently, and they are often responsible for coordinating the actions of multiple cells. One such convergent pathway involves cells communicating with endometrial cancroid cells. These cells are responsible for the growth and spread of cancer, and they use convergent signaling pathways to communicate with other cells in the tumor. In the previous section, details of the RAS pathway show the activation of different signaling molecules. RAS proteins are important for growth and survival in many cells, but they can also help cancer cells to spread (15). RAS proteins help the cells to break down their proteins and cells, whereas RAS inhibitors are anticipated to prevent the spread of tumors (16). Vasoconstriction, sodium–water retention, elevated arterial blood pressure, and enhanced myocardial contractility are the overall effects of RAAS activation, and they together increase the effective circulation volume. This section connects the convergent pathways with the RAS pathway to understand the inhibition of the apoptosis process with cell proliferation.

### 3.1 TNFα signaling pathway

**Figure 2:**
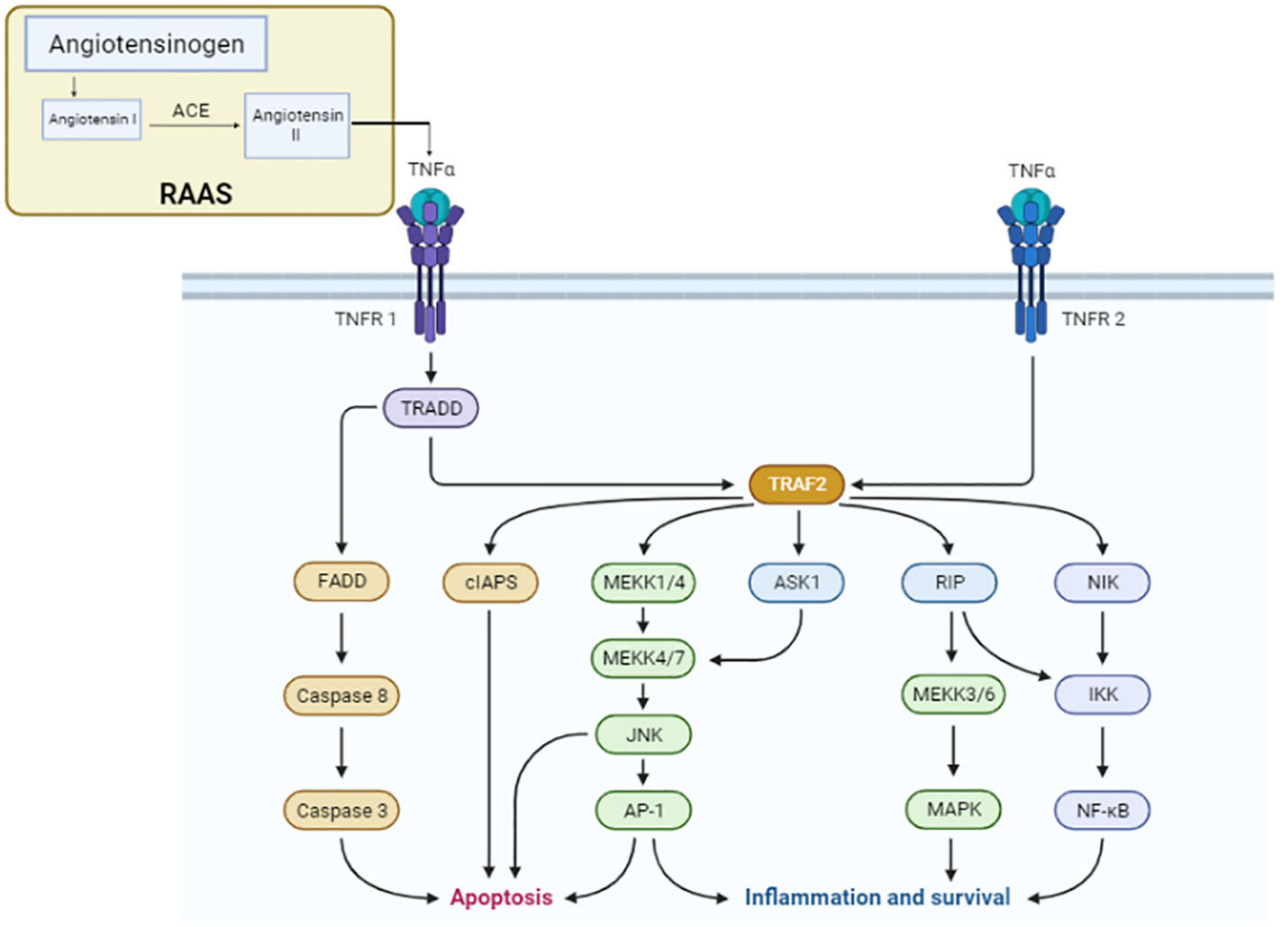
TNFα signaling pathway. The components of the renin–angiotensin system pathway involved in the conversion of angiotensin I to angiotensin II via ACE activate the TNFα receptor for the progression of the cascade of the signaling pathway for cancer development. In the TNF signaling pathway, TNFR1 transmits inflammatory signals by recruiting RIP1 and TRAF2 and apoptotic signals by recruiting FADD and caspase 8. To transmit signals related to inflammation, TNFR2 binds TRAF1 and TRAF2. Pro-caspase 8, the long isoform of FLICE-like inhibitory protein, and non-ubiquitylated receptor-interacting serine/threonine-protein kinase 1 (RIPK1) are additional apoptosis signaling pathways by which TNF can cause cell death (FLIPL).

Inflammation is crucially controlled by the TNF signaling system. The TNFα signaling pathway is composed of several steps, which are illustrated in **Figure 2**. This pathway mediates the stimulation of cytokines like TNF by pro-inflammatory inputs from cells like bacteria or viruses (17). These cytokines then initiate the activation of cells in the immune system, which, in turn, can lead to the inflammation of tissues. For the first time, the activation of the RAS in animals with a specific overexpression of TNF demonstrates that the RAS and proinflammatory cytokines have a functionally important cross-talk in endometrial cancer (18). The primary effect of the renin–angiotensin system is that angiotensin II majorly regulates the release of aldosterone. Additionally, ANG II interacts with the TNF signaling pathway to specifically enhance the functional tissue factors of cell surface activity (19). Aldosterone release is regulated by angiotensin II (Ang II), the primary effector of the RAS. Moreover, it was investigated that AngII and TNF- might interact to specifically enhance the functional tissue factor (TF) cell surface activity. The activation of TNF, in murine hepatoblastoma cells and juxtaglomerular cells, expresses more angiotensinogen and renin mRNA. The first step involved in the activation of TNF receptors, located on the cell surface, is mediated by the primary component of RAS, angiotensin II, as discussed in **Figure 2**. This activation results in the release of the cytokines TNFα and IL-1β. Adipose cells, TAM, or tumor cells show the TNFα signaling pathway, which is responsible for the expression of aromatase. Thus, the upregulation of aromatase via the TNFα signaling pathway promotes the growth of cancer cells. Interestingly, TNFα boosts the production of estradiol, which binds to ER and encourages the growth of luminal cancer cells (20). Activated T lymphocytes, macrophages, and natural killer (NK) cells are the major producers of TNFα. By promoting psoriasis’s mobilization of inflammatory cells, TNFα promotes the NF-B signal pathway-mediated keratinocyte proliferation and anti-apoptosis, ultimately resulting in the development of a micro-abscess (21). The TNFα signaling pathway is crucial for many pathological and physiological processes, including the control of immunological responses, the generation of inflammation, and cell proliferation, differentiation, and death.

### 3.2 TGF-β signaling

**Figure 3:**
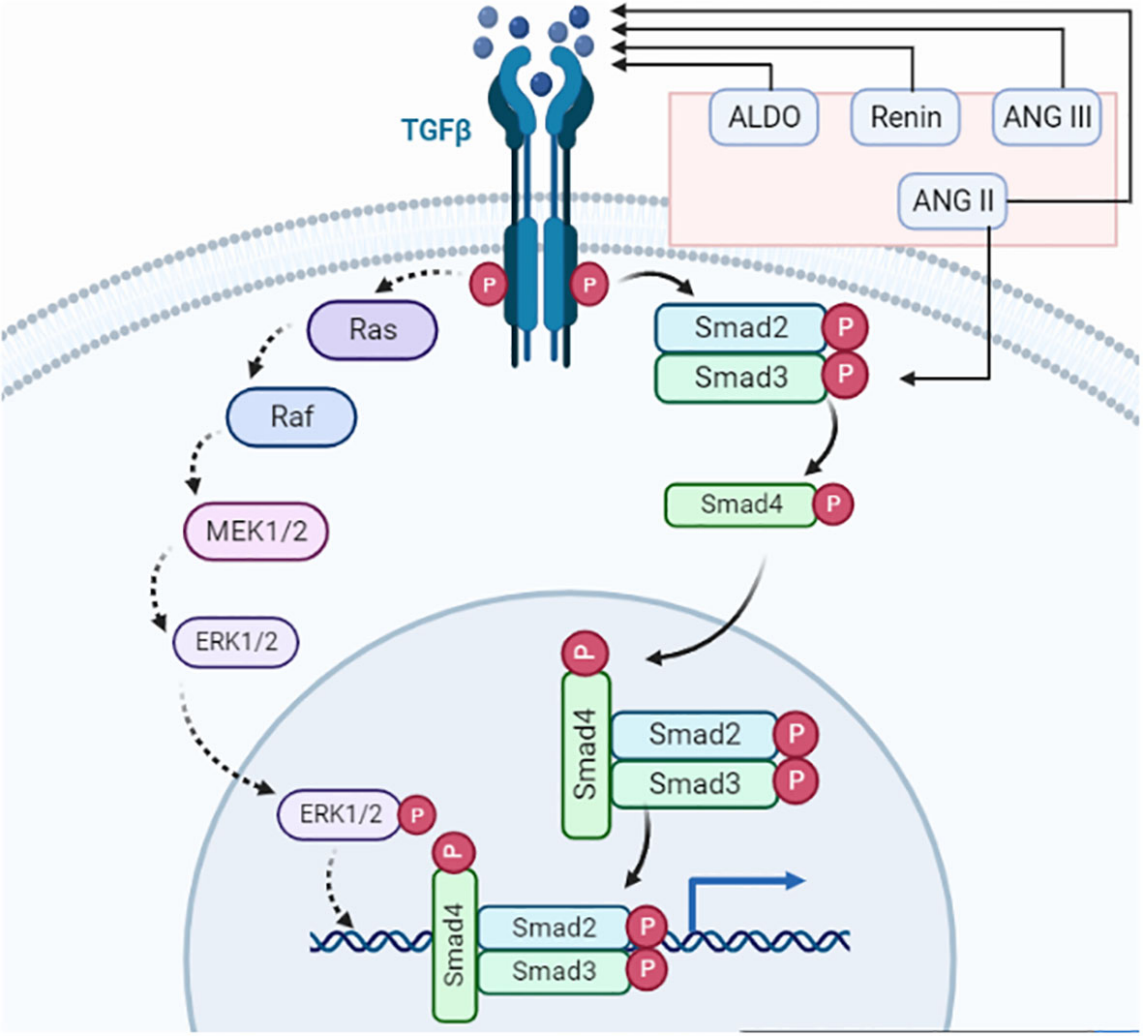
TGF-β signaling. The renin–angiotensin system primary component ANG II activates TGF-β and Smad3. TGF-ligand and TGF-receptor bind to form a complex. The receptor-induced phosphorylation of R-Smads leads to an interaction with cytoplasmic Smad2/3. Phosphorylated Smads combine with Smad4, which facilitates the transport to the nucleus where it connects with multiple transcription factors for transcriptional activities. Smad complexes start a negative loop, which makes Smad7 stop R-Smads from getting any more phosphorylation. TGF-receptors also phosphorylate TAK1 and CREB, which are involved in neuronal differentiation, axonal growth, cell cycle progression, and antidepressant effects.

**Figure 4:**
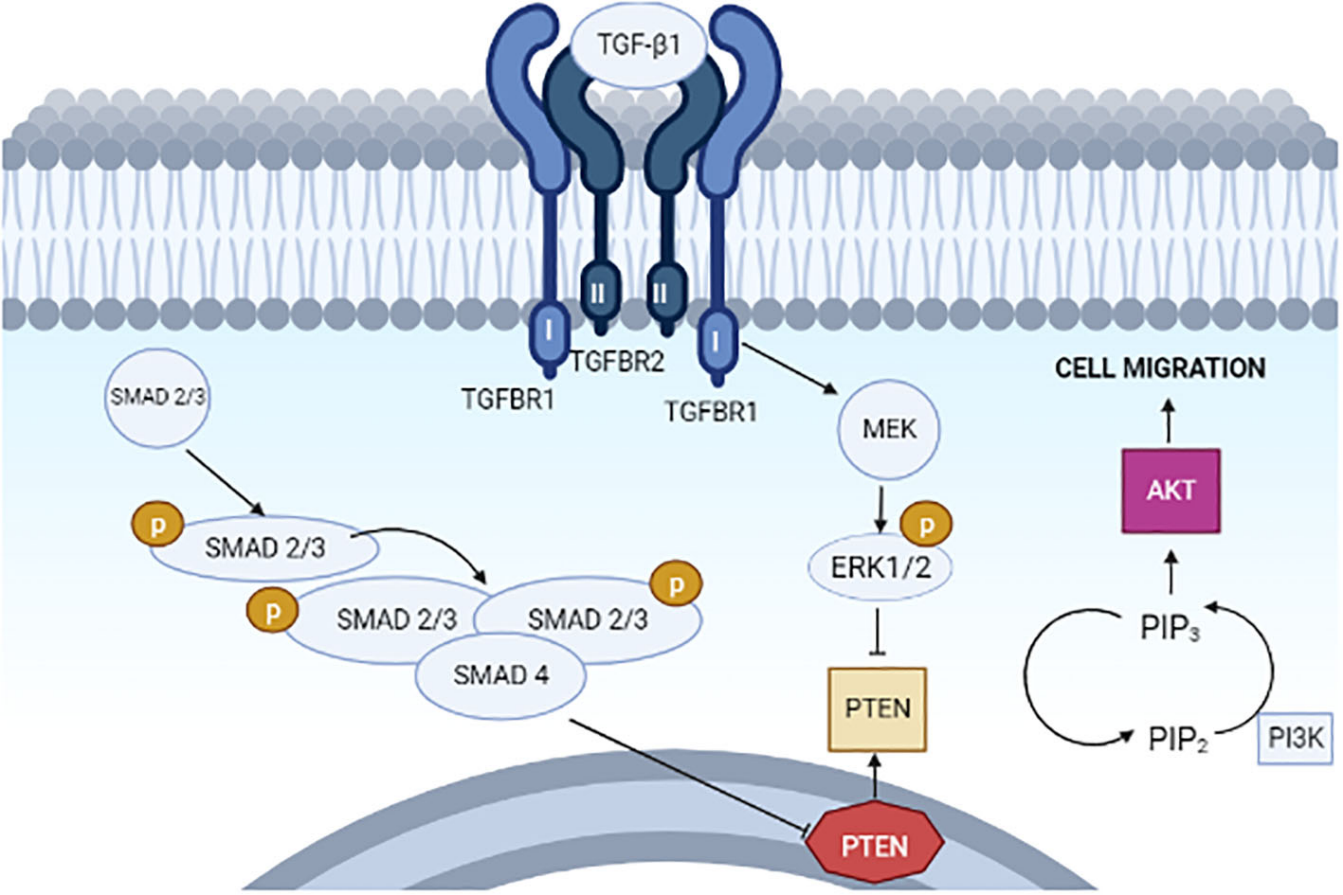
Proposed model for the actions of TGF-β1 on PTEN, PI3K–AKT signaling, and cell migration in type II endometrial cancer cells.

The TGF family of proteins is a sizable set of proteins with a shared structural makeup that functions in a variety of biological activities. The TGF proteins, which are made up of several amino acids and are split into two groups called TGF-1 and TGF-2, are known as a family of proteins. TGF proteins are essential for the growth of tissues, the formation of the embryo, and the preservation of the body’s proper cell balance (21). Independent of blood pressure, the RAAS can increase TGF-β signaling via ANG II. Therefore, angiotensin II enhances the expression of TGF-β receptors, further amplifying the effects of TGF-β1. In general, elements of the RAAS can increase the concentration of TGF-β by affecting several pathways. Through a series of processes, angiotensin II (ANG II) increases TGF-β receptors and induces the production of TGF-β in the kidney. Thrombospondin-1 is stimulated by ANG II through the p38-mitogen-activated protein kinase and c-jun N-terminal kinase signaling, which increases the release of active TGF-β1 from the dormant latent complex (22)—for instance, renin via the (pro) renin receptor, angiotensin II via the AT1 receptor, and aldosterone via the mineralocorticoid receptor all boost TGF-β-1 expression (18). Without triggering TGF-β, ANG II can directly phosphorylate Smads. Mesangial cells and renal interstitial fibroblasts both express more TGF-β1 mRNA under the influence of ANG III which binds to AT1 receptors. Moreover, in these cells, this peptide promotes the production of extracellular matrix in the TGF-β pathway. TGF-β receptor is present in the cell membrane which activates the Ras/Raf/MEK/ERK pathway and Smad2/3 pathway for managing the tumor response in an organ. Finally, PI3K signaling is regulated and the action of apoptosis is inhibited (**Figure 3**). Moreover, TGF-β1, an isoform of TGF-β, inhibits phosphatase and tensin homolog (PTEN) transcription by binding to a combination of type I and type II receptors. Thus, phosphorylating and activating receptor-regulated SMAD2/3 proteins, which then bind to common SMAD4 and move into the nucleus (23). The ligand–receptor complex then activates MEK, which phosphorylates and activates ERK1/2, thereby causing the suppression of PTEN protein post-transcriptionally. The TGF-β1-induced type II endometrial cancer cell migration is crucially mediated via PI3K–AKT signaling, which is enhanced by PTEN downregulation (**Figure 4**) (23).

### 3.3 RAAS and different receptors

**Figure 5:**
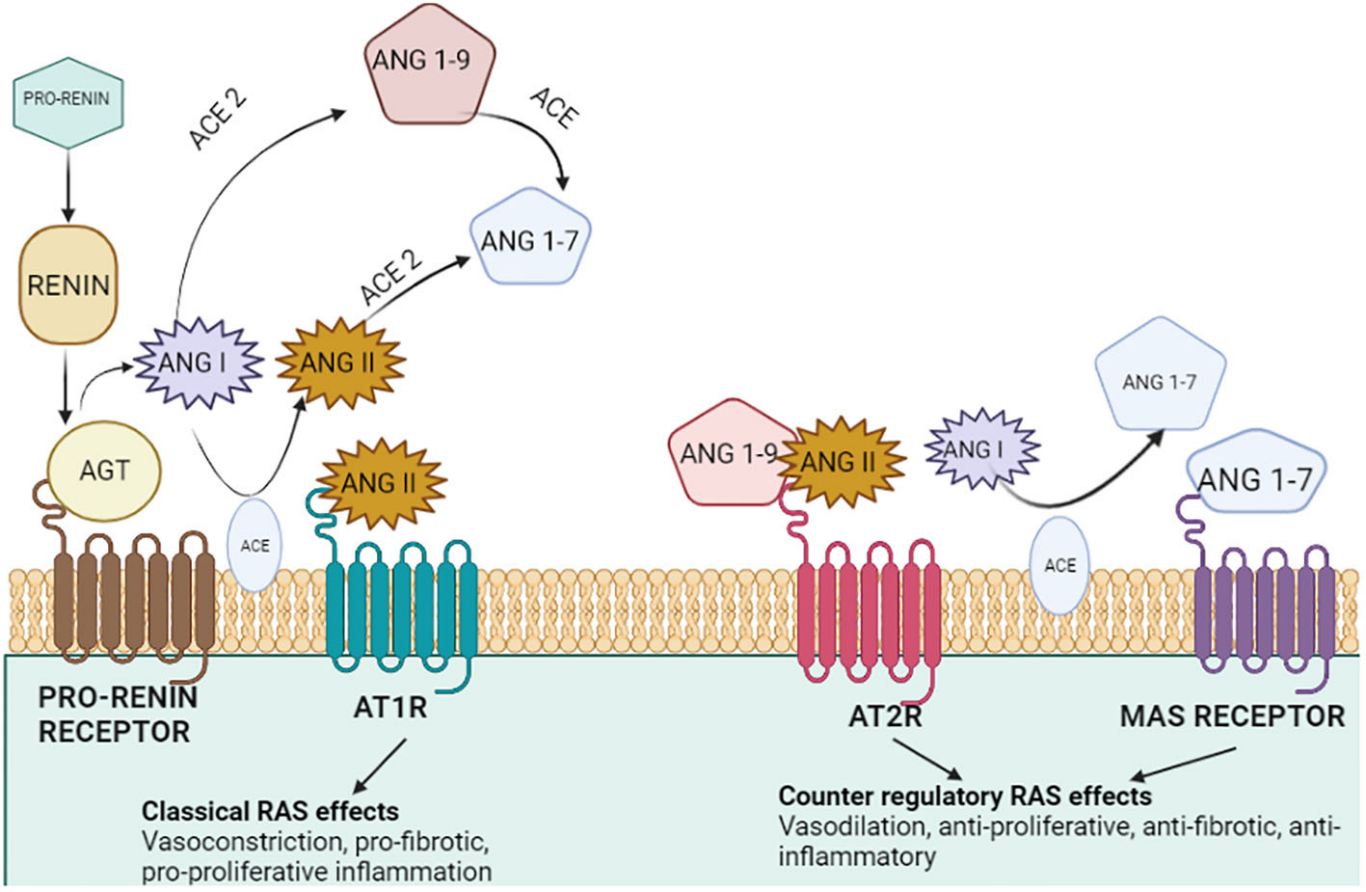
Tissue renin–angiotensin system in endometrial cancer. The physiologically active AngII may be produced with greater ACE1 abundance. The upregulation of the pro-angiogenic and pro-proliferative factors works together to boost the activation of the (P)RR and AGTR1-mediated intracellular signaling cascades, which would then drive the synthesis of TGFB1 and PI3KR1 and aid in the development of tumors. Through vascularization, the presence of MAS1 and increased ACE2 may promote tumor formation (24).

A counter-regulatory mechanism includes neprilysin (NEP) and angiotensin-converting enzyme 1 (ACE1) for the formation of different angiotensinogens. Angiotensin (1–7) is formed via the cleavage of AngII by ACE2 and NEP, whereas Ang (1–9) is formed by ACE2 which activates AT2R, causing natriuresis. Additionally, angiotensin 1–7 binds to the proto-oncogene Mas receptor, causing vasodilation and antihypertensive and anti-fibrotic properties (**Figure 5**) (24). ANG II and ANG (1–7) are both important to induce nitric oxide formation via phosphatidylinositol 3-kinase (PI3K) which restricts cell proliferation (25). Angiotensin II (Ang II), a primary RAS component produced via the conversion of ANG I by ACE, induces angiogenesis and cell growth by binding to its receptor—angiotensin II type 1 receptor (AGTR1) (26). Moreover, the ACE2/Ang (1–7)/MasR pathway, another RAS route, competes with the Ang II/AGTR1 pathway, leading to angiogenesis. Furthermore, the binding of prorenin to the (P)RR causes cell proliferation and tumorigenic intracellular communications which are predominantly independent of Ang synthesis. Tumor growth and drug resistance are both influenced by the activation of the PI3K/AKT/mTOR pathways. Prorenin binding to (P)RR has been shown to activate the ERK1/2 signaling pathway and boost the VEGF protein level which is responsible for VEGFR activation in human diabetic retinopathy and several tumorous cancers enhancing angiogenesis. Hence, a positive correlation between (P)RR on VEGFR activation in the progression of cancer such as lung cancer, breast cancer, colorectal cancer, and endometrial cancer through angiogenesis via the PI3K pathway can be elucidated (27).

**Figure 6:**
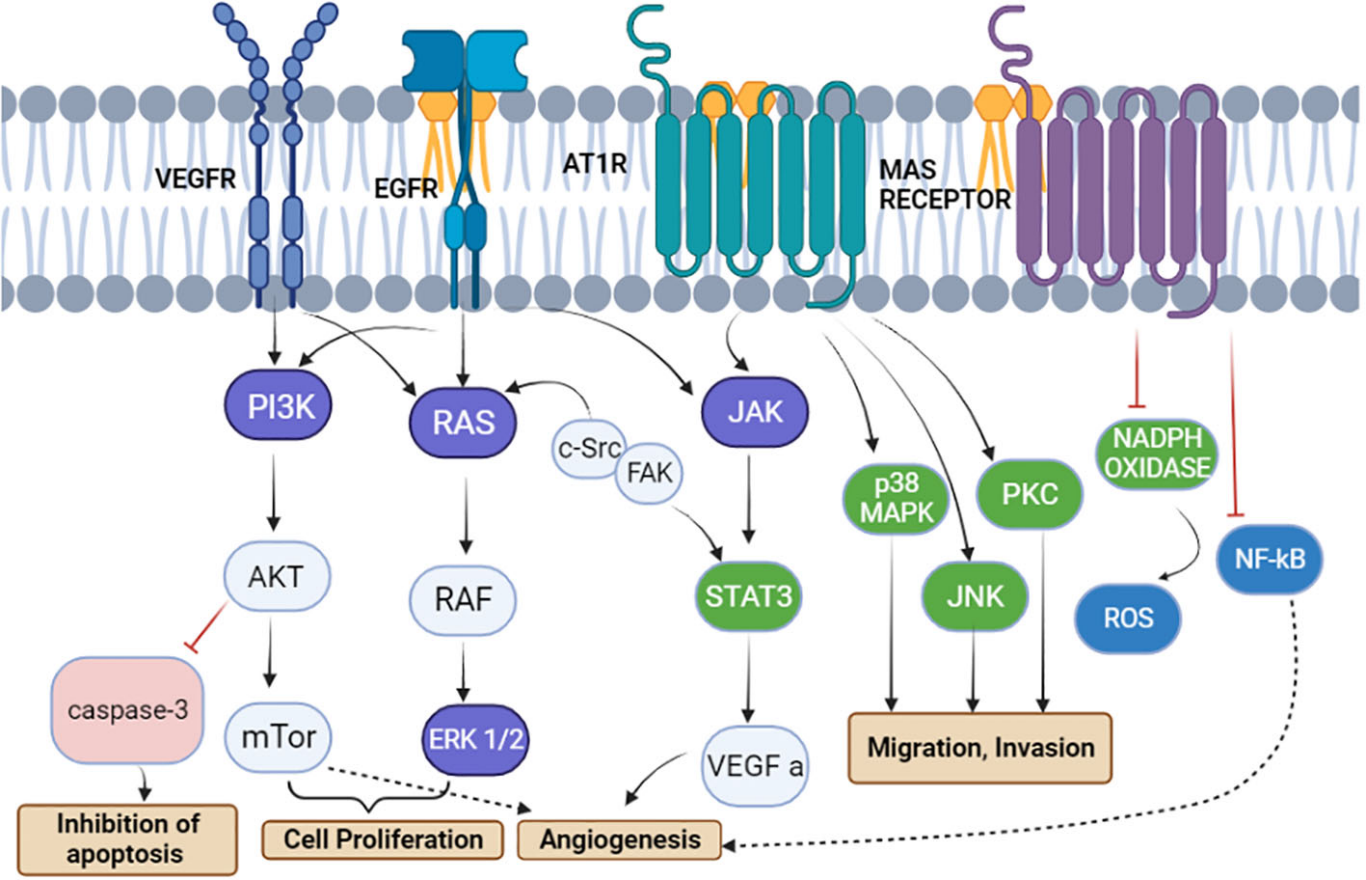
The renin–angiotensin system induces signal transduction pathways that are linked to cell division, migration, invasion, apoptosis suppression, and angiogenesis. Angiotensinogen type 1 receptor activation leads to a cascade of signaling pathways involving JAK, p38MPK, PKC, and JNK, causing the migration and invasion of tumor cells. PRR activates the vascular endothelial growth factor receptor (VEGFR) and epidermal growth factor receptor to activate the PI3K/AKT/mTOR and Ras/Raf/ERK pathways, respectively, for cell proliferation. Moreover, the inhibition of caspase 3 via the AKT pathway leads to inhibition of apoptosis, whereas VEGFR and MAS receptors are involved in angiogenesis (24).

Endothelial cells and fibroblasts, which are significant elements of the tumor microenvironment, as well as tumor cells themselves, can produce and express RAS components that support angiogenesis. Ang II/AT1R, Ang II/AT2R, and Ang 1–7/MAS receptor axis signaling effects have primarily been investigated in tumor cells. The Ang (1,7)-MAS receptor and Ang II-AT2R pathways are believed to block many of the cellular actions of the Ang II–AT1R axis, in contrast to Ang II/AT1R, which mediates several psychopathic events associated with activated RAS, such as increased expression of cell proliferation, a decrease in apoptosis, motility, migration, invasion, and angiogenesis. Interestingly, PI3K signaling, a critical pathway for cell growth and metabolism, initiates cell proliferation and inhibits the activation of caspase 3 responsible for the activation of apoptosis (24). PI3K activity triggers the activation of numerous proteins, including those involved in the cell’s energy production and metabolism (28). PI3K signaling is essential for cell growth and survival—for example, when cells are damaged or stressed, PI3K signaling can help them to recover (29), whereas other signaling pathways such as JNK and PKC initiate migration and invasion as per **Figure 6**. In addition, the cell’s reaction to chemicals and cytokines is one of the critical processes that PI3K signaling controls.

## 4 AngII, AT1R, and AT2R and endometrial cancer

The pathogenesis of endometriosis is based on multiple data sets of genes which mainly include the AGTR1 gene responsible for encoding angiotensin II receptor type 1 (AT1R). The AngII possesses high binding affinity with two specific receptors, i.e., AT1R and AT2R (30). AT1R, involved in regulating the RAS, has a more prominent role in the development and progression of endometrial cancer due to the overexpression of angiogenic factors which affect the progression, proliferation, and apoptosis of EC cells (31). Apart from endometrial cancer, AngII/AT1R has shown a correlation with several other types of cancers such as breast, cervical, prostate, and lung cancer, thereby attributing to the overexpression of the pro-angiogenic and proliferative AngII/AT1R arm of the RAS (6). Additionally, targeting the AT1R proves to have a successful outcome, whereas several AT1R-blocking drugs such as losartan and telmisartan experimented through *in vitro* studies have shown a positive inhibition of EC cell growth, thus also blocking AngII actions (3). Interestingly, silencing of AT1R expression curbs the migration and invasion ability of EC cells. However, the complex role of AngII in the development of EC does not make silencing of angiotensin receptor 1 (AT1R) a successful approach to prevent the progression of endometrial cancer (31).

Angiotensin II, a potent RAS-derived vasoconstrictor peptide involved in tumor angiogenesis, is a prognostic predictor of endometrial endometroid adenocarcinoma along with AT1R, VEGF, and human A-LAP, a potential AngII degradation marker (4). Chidambaram et al. studied the positive correlation between RAS and the menstrual cycle. They noticed that RAS components such as AngII showed elevated levels in the luteal phase among the various phases of the menstrual cycle. Moreover, with the help of the immunostaining method, the localization of AngII, AT1R, and VEGF in corpora lutea indicates the role of AngII in luteal function (30, 31), whereas Ahmed et al. noticed the difference in the functioning of AngII, especially in the proliferative and secretory phases of the menstrual cycle, thereby suggesting the role of AngII in the regeneration of the endometrium after menstruation. Significantly, Piastowska-Ciesielska et al. noticed a high expression of AT1R and AT2R in the G1 stage of endometrial carcinoma, whereas a low expression was observed in the G3 stage. Ishikawa cells, i.e., EC cell lines, were reported to show a correlation between their cell cycle progression, proliferation, and expression of AT1R (31). Apart from the individual effect of AngII on endometrial cancer, the combined effect of microRNA 155 on inhibiting the translation of AT1R decreases the proliferation of EC cells. Thus, the AT1R-blocking drug, losartan, together with anti-mRNA-155, showed a synergistic effect in the reduction of the proliferative effect of AngII in endometrial cancer (31).

## 5 Angiotensin-converting enzyme 1 in endometrial cancer

The twin functions of the ACE, which converts dormant AngI to active AngII and degrades active bradykinin (BK), are well known and play a significant part in the regulation of blood pressure. The conversion of decapeptide angiotensin I to octapeptide angiotensin II is catalyzed by the angiotensin-I-converting enzyme (ACE), a monomeric, membrane-bound, zinc- and chloride-dependent peptidyl dipeptidase. The angiotensin-converting enzyme converts angiotensin I to angiotensin II in the blood. Blood arteries are immediately affected by angiotensin II, which causes them to contract and the blood pressure to rise. ACE1 is an enzyme that helps the body process angiotensin, a hormone that helps to control blood pressure. ACE1 is also responsible for breaking down other chemicals that can cause heart disease. ACE1 is involved in the process of making cholesterol. Researchers have shown that ACE1 is linked to the development of endometrial cancer (32). In Caucasian and Asian groups, the I/D polymorphism of the ACE1 gene has received extensive research attention about cancer. Based on the evidence from several studies, researchers investigated the impact of ACE1 I/D polymorphism on the most prevalent benign pelvic tumor in women all over the world. The increased expression of AGTR1, ATP6AP2, and ACE1, crucial components of the RAS’s pro-angiogenic/proliferative arm, raises the possibility that the RAS is involved in the development and progression of endometrial cancer (33). Consequently, drugs that are now in the market that block the RAS and are used to treat high blood pressure may one day be utilized to treat endometrial cancer. Selective AT1 receptor blockers and ACE inhibitors (ACEIs) are the two main groups of medications that target the RAS (ARBs). Even though both medication classes have angiotensin II as their target, the variations in their methods of action have an impact on how they affect other pathways and receptors, which could have therapeutic ramifications. By preventing the conversion of angiotensin I to angiotensin II, the ACEIs lessen the activation of the RAS and the AT1 and AT2 receptors. The pathogenic effects of angiotensin II, including vasoconstriction and other processes that raise the blood pressure as well as cause vascular hypertrophy, endothelial dysfunction, atherosclerosis, inflammation, and apoptosis, are mostly mediated by angiotensin II type 1 receptors. Angiotensin II reactivation has been linked to chronic treatment, even though acute treatment with ACEI decreases circulating angiotensin II to insignificant levels.

According to research done on endometrial cancer patients, the tumor tissues had higher amounts of AT1R, ACE1, and ACE2 mRNA than the surrounding non-cancerous tissues. It is unknown if ACEIs have a similar impact on the expression of ACE2, ACE1, and TMPRSS2 in the human endometrium. Hence, restoring the balance using ACE inhibitors is one of the main treatment approaches for halting the progression of the disease. The glandular epithelium (GE) and luminal epithelium were the primary sites of ACE1 protein expression (26). Rather than cancer, ACE1 and ACE2 had immense activity on the immunological imbalance which can lead to deadly diseases—for instance, there is a connection of imbalance in the ACE1 and ACE2 expression on the progression of COVID-19 which causes deadly long-term issues. By using Western blotting, the expression of the proteins ACE2, ACE1, and TMPRSS2 was examined and normalized to that of actin or tubulin. The expression of TIMPRSS2, ACE1, and ACE2 transcripts in Ishikawa cells exposed to a range of ACE1 and ACE2 inhibitor doses (0.3–30 M) for 24 h was also observed.

According to a previous study, ACE1 inhibitors decrease AngII production and Ang-(1–7) metabolism because ACE2 increases AngII metabolism. According to a recent study, for the treatment of chronic illnesses, people taking ACE1 inhibitors may also be at risk of contracting SARS-CoV-2 infection because of the increased expression of the ACE2 receptor (34). ACE1, ACE2, and TMPRSS2 transcripts and proteins are expressed in the human endometrium. For *in vitro* research on endometrial receptivity and embryo implantation, the commonly utilized human-sensitive endometrial Ishikawa and RL95-2 cells both express ACE1, ACE2, and TMPRSS2 transcripts and proteins. Since the ACEIs used in the current study did not affect the expression of ACE1, ACE2, and TMPRSS2 or the attachment of the spheroid to the Ishikawa cells, this suggests that using ACEIs to treat diseases may not have an impact on endometriosis and subsequent embryo implantation (35).

The RAS pathway involves a series of proteases that result in the production of several bioactive compounds. Angiotensinogen is broken down by renin, which is secreted by the juxtaglomerular cells of the kidney and primarily released by the liver, to create decapeptide angiotensin I (Ang I). Angiotensin-converting enzymes (ACE), which are expressed by endothelial cells in several organs including the lung, kidney, heart, and brain, change AngI into Ang II. The most important RAS pathway chemical, Ang II, works by activating the G-protein-coupled receptors AT1R and angiotensin II receptor type 2 (AT2R) (36).

The importance of ACE1 in endometrial cancer has been well documented. ACE1 is a key enzyme that helps control cell growth and division. When ACE1 is absent or poorly functioning, cells can grow unchecked and form tumors. ACE1 is also critical for the elimination of damaged cells. When cells are damaged, they can release inflammatory signals that can spur the growth of tumors. If ACE1 is absent or impaired, these inflammatory signals can build up and lead to the development of endometrial cancer. The role of ACE1 in endometrial cancer has been well studied, and the importance of this enzyme has been highlighted in many research studies.

Compared to limiting ACE, some researchers also think that blocking the AT1R may lessen the inflammatory mediators’ reactions and decrease acute lung injury. Particularly, the ACE inhibitor captopril significantly decreased lipopolysaccharide, decreased the release of tumor necrosis factor and interleukin 6, decreased the ratio of AngII to Ang 1–7, and attempted to reverse the higher ratio of ACE to ACE2 in animal models of lung injury (37). Angiotensin I (AngI), which is traditionally created by the protease renin acting on liver-derived angiotensinogen, is then converted to AngII by the angiotensin-converting enzyme 1 (ACE1). Angiotensin II (AngII) can then attach to two separate GPCRs called AT1R and AT2R, which are responsible for this hormone’s physiological effects. Interestingly, the cardiovascular-regulating activities of AngII are connected to AT1R activation (38). The communication that follows mediates the vasoactive effects of AngII. Nevertheless, excessive AngII : AT1R signaling may result in the clinical diseases listed above.

## 6 ACE2 and endometrial cancer

The whole RAS cascade stimulates the migration of cells, their proliferation, and angiogenesis in healthy endometrium. In the same way, if over-activated or over-expressed, it can lead to abnormal cell growth, setting the hallmark for endometrial cancer (39).

Whenever loss of blood occurs, which can be due to multiple reasons, the blood pressure drops instantly, and pressure-measuring smooth muscles lining the renal afferent arteriole, called Polkissen cells, detect this variation in blood pressure, and this leads to a series of events with the target of being able to maintain the blood volume and blood pressure (40). A pivotal component of this cascade is angiotensin-converting enzyme 2 (ACE2), which facilitates vasoconstriction and maintains hydro-salinity balance. Its distribution in the female reproductive system also suggested that it may have a role in controlling follicle growth and ovulation as well as regulating luteal angiogenesis and degeneration (41).

A significant death rate is associated with one kind of uterine corpus endometrial carcinoma (UCEC), an endometrial epithelial malignant tumor. Its dependency on the hormone estrogen serves as the basis for division. Despite the limited occurrence of non-estrogen-dependent tumors, the prognosis, in this case, is bad due to the high malignancy. Additionally, ACE2 provided a positive prognosis (26).

### 6.1 Structure, function, and localization of angiotensin-converting enzyme 2

**Figure 7:**
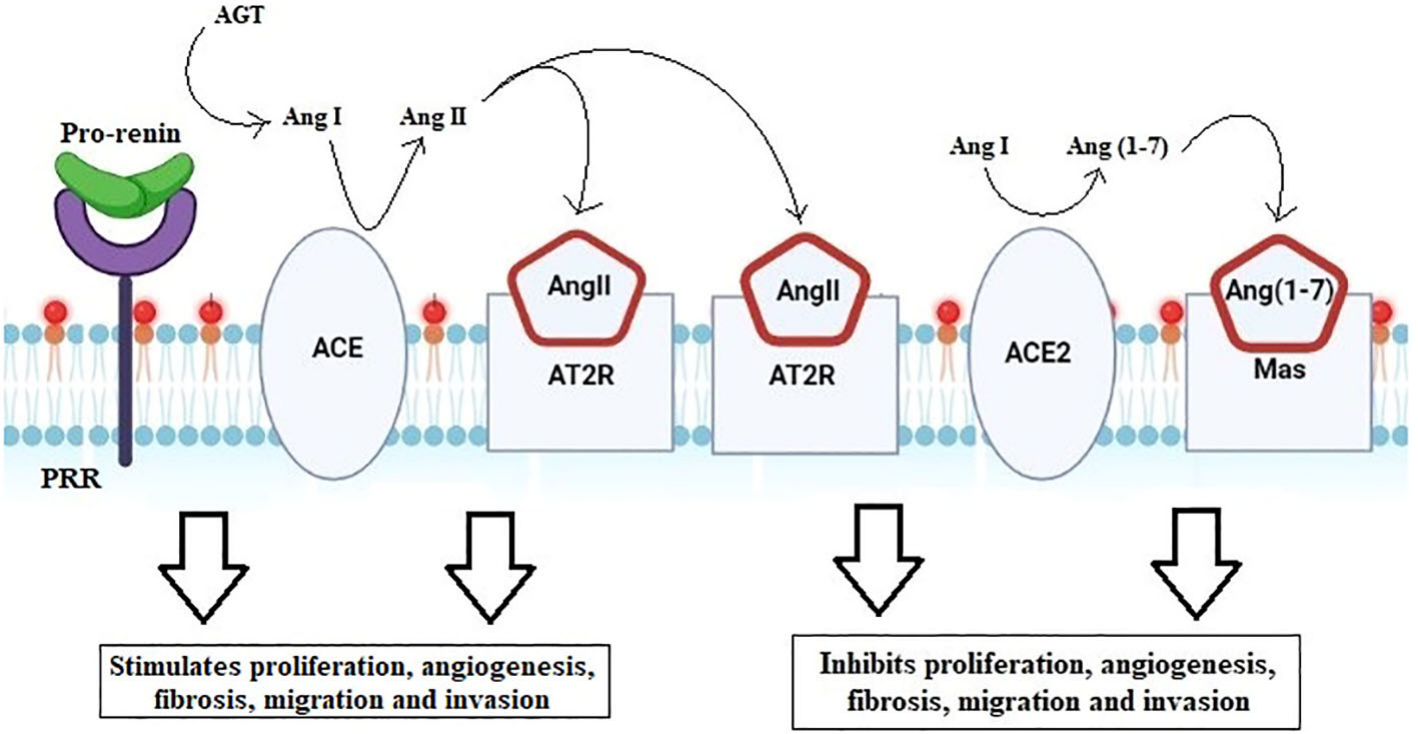
Renin–angiotensin system cascade. Activation of pro-renin by PRR forms angiotensin from angiotensinogen. ACE then converts AngI to the biologically active AngII. Angiotensin binds to either the angiotensin II type 1 receptor (AT1R) or the angiotensin II type 2 receptor (AT2R). The downstream pathway run, by binding to AT1R, stimulates angiogenesis, fibrosis, migration, and invasion, whereas binding to AT2R acts antagonistically, blocking proliferation, angiogenesis, migration, and invasion.

The open reading frame of the ACE2 transcript encodes an 805-amino-acid polypeptide (41). The extracellular catalytic region of the zinc metalloprotease ACE2 is 42% homologous to the N-terminal catalytic domain of ACE. Given that it is a protease, the enzyme can cleave angiotensin II into angiotensin (1–7) that then displays the necessary functions, as explained in **Figure 7** (26, 39, 41).

The information at hand suggests that ACE2 is broadly expressed in the uterus, ovary, vagina, and placenta in addition to being localized in the lung epithelium. The oocyte and ovary levels were discovered to be relatively high (42). After examining information from HP Atlas and Gene Cards, the existence of ACE2 in the uterus and vagina was established (41).

In the menstrual cycle’s proliferative stage, stromal cells and endometrial epithelium were both reported to express ACE2. However, it has been observed that expression rises throughout the secretory phase. In addition to oocytes from immature rat ovaries, the presence of ACE2 in stroma and granulosa cells has recently been discovered (43). Additional research has revealed that it exists in the granulosa and theca cells of cattle. In the human placenta, there was evidence of increased ACE2 expression (44). Its expression in primary and secondary placental villi endothelium and vascular smooth muscles, syncytiotrophoblast, and cytotrophoblast was found. Additional evidence of ACE2 was discovered in the atrial and venous endothelium as well as the smooth muscles of the umbilical cord, where ACE2 first manifests in early pregnancy. Moreover, ACE2 expression in the placenta is higher than that in the lungs (41).

As far as the function of ACE2 is put into consideration, it balances AngII and Ang in a synergistic manner (1–7) and later facilitates follicle development and ovary maturation via inducing steroid secretion. Studies suggested its contribution to follicular atresia, enhancing ovulation, maintaining corpus luteum progression, and promoting the production of estradiol and progesterone (41, 45).

Evidence points to its function in the progesterone-mediated differentiation process known as stromal cell decidualization, which primes uterine stromal cells for implantation. The same was demonstrated by the reduction of the decidualization response when ACE2-targeting siRNA was transfected into human endometrial stromal cells (43).

Additionally, AngII can induce angiogenesis, fibrosis, migration, invasion, and cell proliferation by acting on the AngII type 1 receptor (AGTR1) and activating growth factors and intracellular signaling pathways. ACE2 can further transform AngII to become Ang (1–7) and later works on its receptor Mas, which causes the earlier route way (AngII/AGTR1) to become antagonistic (46).

Vascular bed and endometrial regeneration are only a couple of the many functions that AngII plays in the endometrium. Through spiral artery constriction, it starts the menstrual cycle. The ideal proportion between AngII and Ang controls endometrial regeneration (1–7). AngII regulates menstrual periods in the endometrium as part of its normal function Dysfunctional uterine hemorrhage and hyperplastic endometrial polyps may be visible if the distribution and amount of AngII in the endometrium depart from normal (47).

Additionally, it was shown that the increased levels of ACE2 and AngII expression were associated with the development and metastasis of endometrial cancer. Increased ACE2 leads to increased conversion of AngI to Ang (1–7), increased Mas, and increased binding of AngII to AT2R, all of which are highlighted mechanisms to counteract the actions of AngII and ATR1 (48, 49). The major ways that AngII, ACE2, and Ang (1–7) operate during pregnancy are via controlling the blood pressure and fetal growth. They work together to preserve healthy uterine physiology in the meanwhile (41). Rat and human cell trophoblast invasions are induced by AngII. In early pregnancy (apoptosis, angiogenesis, and growth) and late pregnancy (uteroplacental blood flow), Ang (1–7) and ACE2 may function as local autocrine/paracrine regulators (41).

### 6.2 ACE2 mRNA expression levels in endometrium carcinoma

**Figure 8:**
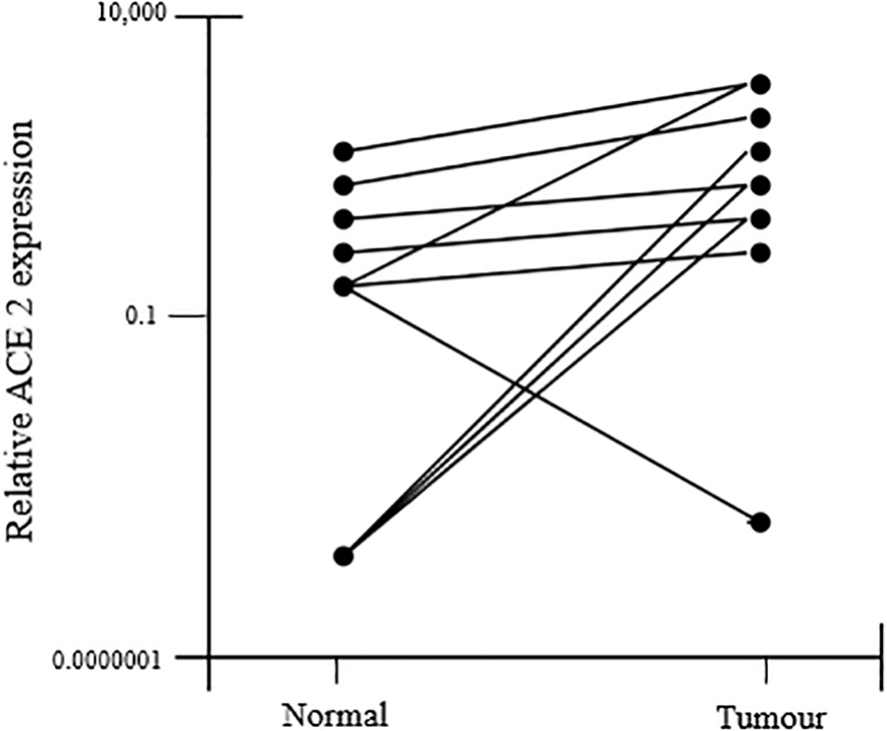
ACE2 mRNA expression was found to increase in tumor tissue as compared to the adjacent non-cancerous tissue.

Immunohistochemistry was used to analyze the expression of downstream RAS pathway targets in endometrial cancer tissue and nearby non-cancerous endometrium, including phosphoinositide-3-kinase (PIK3R1), plasminogen activator inhibitor-1 (SERPINE1), VEGFA, and transforming growth factor beta 1 (TGFB1). Their expression was shown to be associated with that of RAS components. Using immunohistochemistry, which considers the protein by-products of all genes, all endometrial tumors and the surrounding non-cancerous tissues were identified. The tumor tissues exhibited significantly higher amounts of ACE1, AGTR1, and ACE2 mRNAs than the non-cancerous nearby tissue, although AGT mRNA did not differ between the malignant and non-cancerous bordering tissue (**Figure 8**) (41, 50).

ACE1, ACE2, AGTR2, AGTR1, and MAS1 protein immunostaining showed the glandular epithelium to be more intense. Additionally, it was found in the stroma, perivascular area, and endothelium. According to the study’s findings, a higher risk of endometrial cancer recurrence is associated with higher levels of AGTR mRNA expression. All of this showed that AGT, by the synthesis of AngII and the overactivation of its receptors, contributes to the development and growth of tumors. The generation of AGT in the liver is estrogen-dependent. Adipose tissue may also express it and secrete it. Therefore, both high estrogen levels and obesity, which have the propensity to enhance AGT production, are substantial risk factors for endometrial cancer, thereby accelerating sickness. High ACEI mRNA levels in endometrial cancers tend to improve their ability to generate AngII.

Additionally, the most effective method for figuring out how the gene ACE2 is regulated is to look for TF binding sites in its promoter. To anticipate the probable TF binding sites, the well-known web-based application Mat Inspector from Geomatics was employed. This aided in the TFs in the ACE2 promoter’s identification. The 1,947-nucleotide-long sequence of the ACE2 promoter, which was taken from PubMed with the NCBI reference sequence ID NG 068141 and is situated on chromosome X, is presented in a FASTA format. For the positive stand of the ACE2 promoter, a total of 51 TFs were identified. Among these 51 TFs, many were found to have high confidence binding sites (match factor greater than 0.9).

Tumor-prompting transcription factor has such a high confidence binding site at ACE2 promoter. Some of these transcription factors are B cell lymphoma 6 (BCL6), Wilm’s tumor protein (WT1), signal transducer and activator of transcription 3 (STAT 3), Ying Yang-1 (YY1), ERG, AREB6, GKLF (or KLF4), and GATA TFs. All these transcription factors have been linked to the emergence of different malignancies, including endometrial cancer (41).

### 6.3 ACE2 and favorable prognosis

Further research was done on the association between ACE2 expression and prognosis in these cancers. No significant relationship between ACE2 expression and the prognosis of breast cancer and squamous cell carcinoma of the head and neck was found in the studies on the same topic (51). On the other hand, kidney renal papillary cell cancer and UCEC had much better prognoses due to high levels of ACE2 expression. Additionally, the relationship between tumor invasion and the prognosis was researched. Here immunological infiltration and ACE2 transcription levels in endometrial cancer were associated (52). The TIMER database has been employed for the same. The findings revealed that the expression of ACE2 was strongly linked with the degree of macrophage immune infiltration in renal papillary cell carcinoma. Similarly, it was discovered that ACE2 and the levels of CD4+ T cell, B cell, neutrophil, and dendritic cell immune infiltration in uterine corpus endometrial cancer are positively correlated (53).

## 7 The (pro) renin receptor in endometrial cancer

The pro-renin receptor is a crucial protein for both healthy cardiovascular and renal function as well as illness. However, P(RR) participates in several other crucial processes; thus, its function is not only restricted to the renal and cardiovascular systems. Over the last 5 years, the pro-renin receptor is irregularly expressed in several malignancies, including endometrial cancer, according to data from ongoing studies (48). It has been established via several trials that P(RR) plays a substantial role in a variety of cancer-causing cells, including endometrial cancer. When compared to healthy neighboring endometrial tissue, it has been demonstrated that P(RR) is over-expressed in human endometrial cancer tissue (54).

It is widely established that P(RR) stimulates angiogenesis and activates several cellular processes, including proliferation and migration, all of which are significant contributors to the onset and development of endometrial cancer (55).

The 350 amino acid sequences that make up pro-renin receptor’s structural makeup make it a multifunctional protein. The N-terminus of this long trans-membrane protein faces the extracellular side of the cell, while the C-terminus faces the cytoplasm. It has a single-membrane-spanning domain. Both the proteases Furin and Adam 19 can cleave P(RR) in the Golgi complex and produce a shortened soluble protein. The soluble portion of the truncated protein is the N-terminus which can then be secreted as P(RR) into the body fluids, whereas the truncated C-terminus remains part of the transmembrane (56). ATP6AP2 which was found on the X chromosome regulates the activity of P(RR) (26).

In conjunction with the pro-renin receptor, the renin–angiotensin system works efficiently in women with a healthy endometrium during pregnancy. In the endometrium, under normal conditions, the main functions of RAS are to promote angiogenesis, cell proliferation, and migration. However, if overexpressed, it can promote irregular cell growth and metastasis, resulting in a classic case of endometrial cancer (57). The P(RR) receptor, as a component of vacuole ATPase, has the functional capability of acidifying the extracellular milieu (58).

In the RAS system, P(RR) plays a notable role. PRR can be bound by ligands like renin and its precursor pro-renin (RR). These molecules become highly active when they meet P(RR), which catalysis the conversion of angiotensinogen to angiotensin (ANG1). The cleaving function of ACE converts angiotensin 1 into angiotensin 2 (angiotensin-converting enzyme) (59). Angiotensin 2 is then produced and binds to its receptor, AGTR1, activating ANG-2 receptor-mediated signal transduction and accelerating processes like angiogenesis and cell division. By boosting the oncogenic factors, the upregulated activity of the ANG2/ANG2 receptor in conjunction with the pro-renin receptor causes cancer (60).

One of the crucial downstream factors generated by P(RR) signaling is TGF, which is thought to be required for the epithelial-to-mesenchymal transition. Since the RAS system activates TGF, there is a close relationship between the two. This TGF has an overexpression in tumor tissues when abnormal signaling is present. This pathway, therefore, suggests that P(RR) promotes cancer spread by activating TGF via RAS (61).

**Figure 9:**
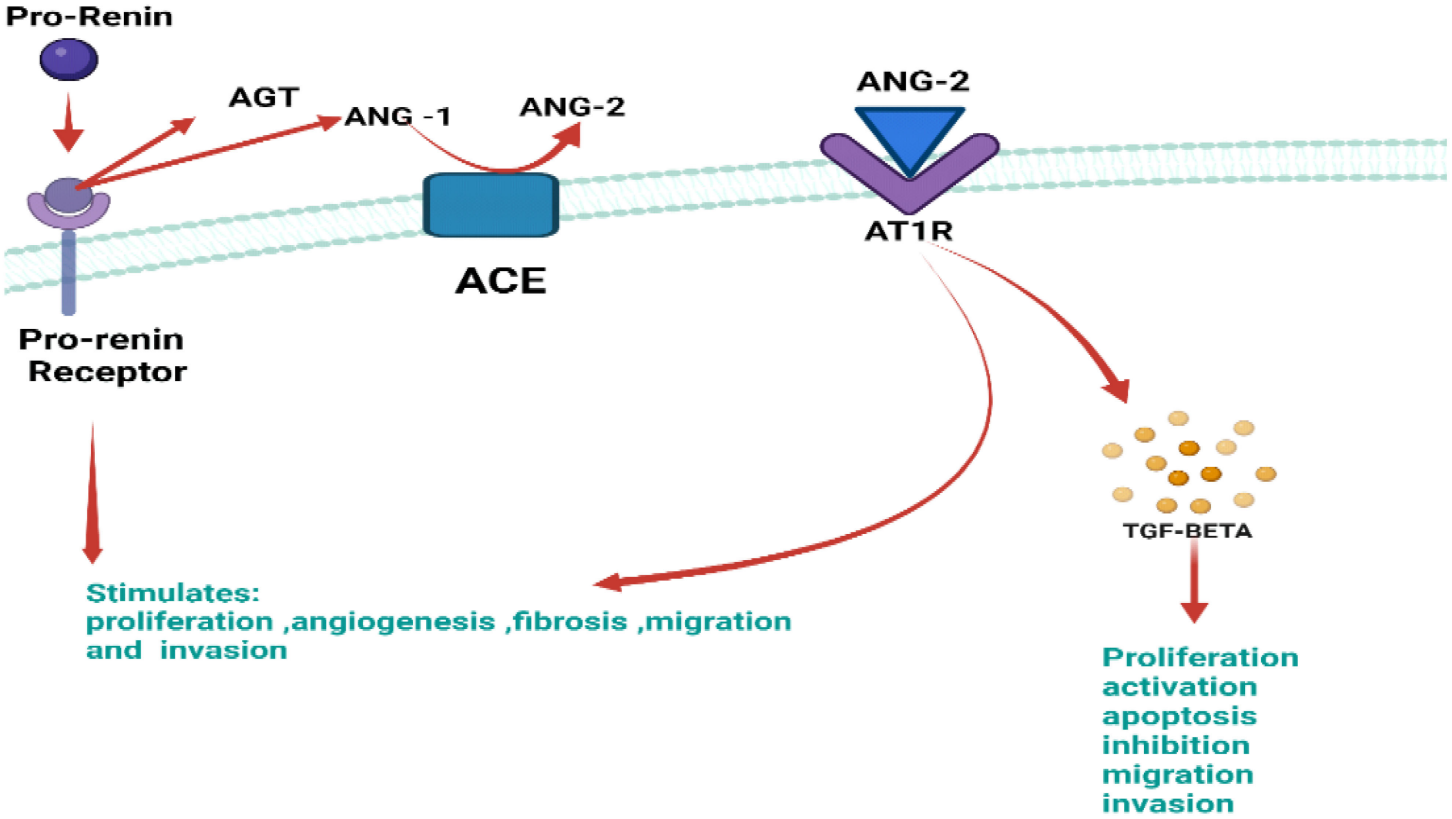
Illustration of the authorized P(RR) signaling with major key components which include pro-renin, ANG-1, ANG-2 AT1R, and TGF-BETA. Abnormally active pro-renin binds to its receptor P(RR), causing the formation of angiotensin-1 from angiotensinogen. Upon interaction of ANG-1 with ACE, it forms ANG-2, which binds to its respective receptor, i.e., AT1R causing TGF-beta formation. This erratic signaling stimulates proliferation, angiogenesis migration, and invasion.

Pro-renin participates in intracellular signaling outside of the authorized route, which is unrelated to ANG2 synthesis and has the potential to be both proliferative and tumorigenic. In non-authorized signaling, P(RR) phosphorylates enzymes such as extracellular signal-regulated kinase 1/2 (ERK1/2) and mitogen-activated kinases (MAPK), which activate TGF. As a direct result of this signaling, P(RR) increases cell proliferation and encourages the unchecked spread and growth of this kind of cancer (**Figure 9**) (62).

When multiple sets of experimental data were seen, the proliferative ability of P(RR) in endometrial cancer was emphasized, highlighting P(RR) as an essential element of endometrial cancer formation. According to Delforce et al., an unbalanced RAS promotes the growth of endometrial cancer. Up to 30 different samples of endometrial cancer and its surrounding normal tissue were examined for protein and mRNA levels of various RAS components. The various RAS components’ protein and mRNA levels were shown to be higher in tumor tissues than in the nearby normal tissue. The protein levels of P(RR) and the mRNA levels of P(RR), AGTR, ACE, and ACE2 in tumor tissues were considerably higher than in the surrounding normal tissue. Another factor closely linked to RAS, i.e., TGF, was found to be abnormally high in tumor tissues. Thus, the results show that pro-renin plays a substantial role in endometrial cancer due to the greater expression of multiple RAS components (**Table 1**) (58).

**Table 1:** Different components of P(RR) signaling that show its effect in cancer, their mRNA expression, and the effect that each component has in cancerous signaling.

S.No.	Pro-renin receptor components	Expression (mRNA and protein)	Outcome	References
1.	P(RR)	Upregulated	Promotes angiogenesis and cell proliferation	(57)
2.	ACE	Upregulated	Enzyme converting ANG1 to ANG 2 and promotes cell proliferation	(57)
3.	AGTR1	Upregulated	Receptor binding RAS components and promoting aggressive cell division	(58)
4.	ANG 2	Upregulated	Binds to the receptor and further activates components that take part in angiogenesis, cell proliferation, and metastasis	(58)
5.	TGF β	Upregulated	Activation of proliferation, inhibition of apoptosis, and helps in the migration of cancerous cells	(59)
6.	MAG protein	Upregulated	Limits the supply of MAX for Myc heterodimerization and negatively regulates Myc-dependent cell processes, thereby transforming tumor cells from a rapidly dividing state to a more regulated state of division	(60)
7.	SLC4A7	Downregulated	Component of P(RR)V ATPase and causes extracellular tumor acidity by changing the activity of PH transporters that facilitate hydrogen ion efflux	(60)
8.	ATP6VOA7 and ATP6VOD7	Downregulated	Assemble the components of P(RR) and contribute to cellular proliferation	(60)

Components of P(RR) such as ACE, P(RR), AGTR1, ANG2, and TGF-beta are upregulated upon the knockdown of the pro-renin receptor. Certain proteins were downregulated.

In this distinctive research, Jacinta et al., Sarah et al., and Riazuddin et al. monitored the mRNA and protein expression of P(RR) in different endometrial cancer cells, namely, Ishikawa cells (grade 1) followed by AN3CA cells (grade 3) and subsequently HEC-1-A cells (grade 2). The results from this study created a contradiction as it demonstrated that carcinoma grade and the mRNA levels of P(RR) had no co-relation, inferring that the mRNA levels of P(RR) functioned independently of the type of cancer (61).

Ishikawa cells were further subjected to siRNA transfection. Some proteins were found to be upregulated, while some were downregulated (the most significant among them being the pro-renin receptor). Notably, the most downregulated protein was found to be the pro-renin receptor, thereby demonstrating the efficiency of the siRNA knockdown procedure (61).

The MAX gene-associated protein (MGA) highlighted an increased expression in the siRNA-transfected Ishikawa cell lines. The functional activity of the MGA protein is a transcriptional activator/repressor which regulates the activity of genes that control cellular processes like proliferation (61). MGA and MYC are the binding partners of Myc-associated factor X (MAX), and upon dimerization of MAX with MGA/MYC, it dictates the transcription of target genes in non-tumorigenic cells. MYC-dependent cell transformation is negatively regulated by MAX and MGA heterodimers (62). Therefore, the over-expression of MGA by the knockdown of P(RR) will curtail the supply of MAX for MYC heterodimerization and stop the MYC-dependent cellular processes from shifting tumor cells from a rapidly dividing state to a more regulated state of division (8).

Extra-cellular tumor acidity is generally interlinked with cancer aggressiveness. Cancers usually show a typical pattern in which the intra-cellular pH is found to be higher than the extracellular pH due to the rapid efflux of H^+^ ions. This rapid efflux could be linked to different modifications (such as expression and activity) in the targeted pH transporters (63). Four proteins involved in the acidification process were downregulated in the siRNA-treated Ishikawa cells which includes isoform 7 of sodium bicarbonate co-transporters (SLC4A7). The other protein that was downregulated in this study included ATP6VOA1 and ATP6VOD1, which are the two subunits required for putting together the V-type proton ATPase in its functional form. As P(RR) is also a component of V-ATPase, the downregulation of P(RR) therefore directly affects the levels of ATP6VOA1 and ATP6VOD1. This provides sufficient evidence that the downregulation of SLC4A7, P(RR) ATP6VOA1, and ATP6VOD1 contributes to proliferation (64).

Overall, it is implicated from the provided data that the components of RAS and P(RR) both, in general, exhibit erratic signaling in endometrial cancer, highlighting unusually high levels in tumor tissues in contrast with the normal tissue which is used as a control. The mRNA and protein levels of P(RR) were higher in targeted cell lines like Ishikawa cells, AN3CA cells, and HEC-1-A cells, but the protein levels were found to be independent of the grade of the tumor. This research could pave the way for various effective treatment methods for endometrial cancer such as siRNA-treated P(RR) or monoclonal antibodies targeted against P(RR). Lastly, knocking down the P(RR) and its related proteins which are generally proliferative in cancerous tissue could prove to be a beneficial and curative strategy for targeting cancer of the endometrium (59).

## 8 The RAS components as a potential therapeutic target in endometrial cancer

One of the factors that have been associated with endometrial cancer is the Ras protein. Ras, which belongs to the family of GTPases, is involved in signaling pathways associated with cell growth and proliferation and regulates diverse cell behaviors. Any over-activation of this protein can bring alterations in the upstream and downstream components of signaling. It plays a vital role in tumor maintenance. Mutation in Ras has been shown by most human carcinomas and hence considered an appropriate target for cancer therapy.

**Figure 10:**
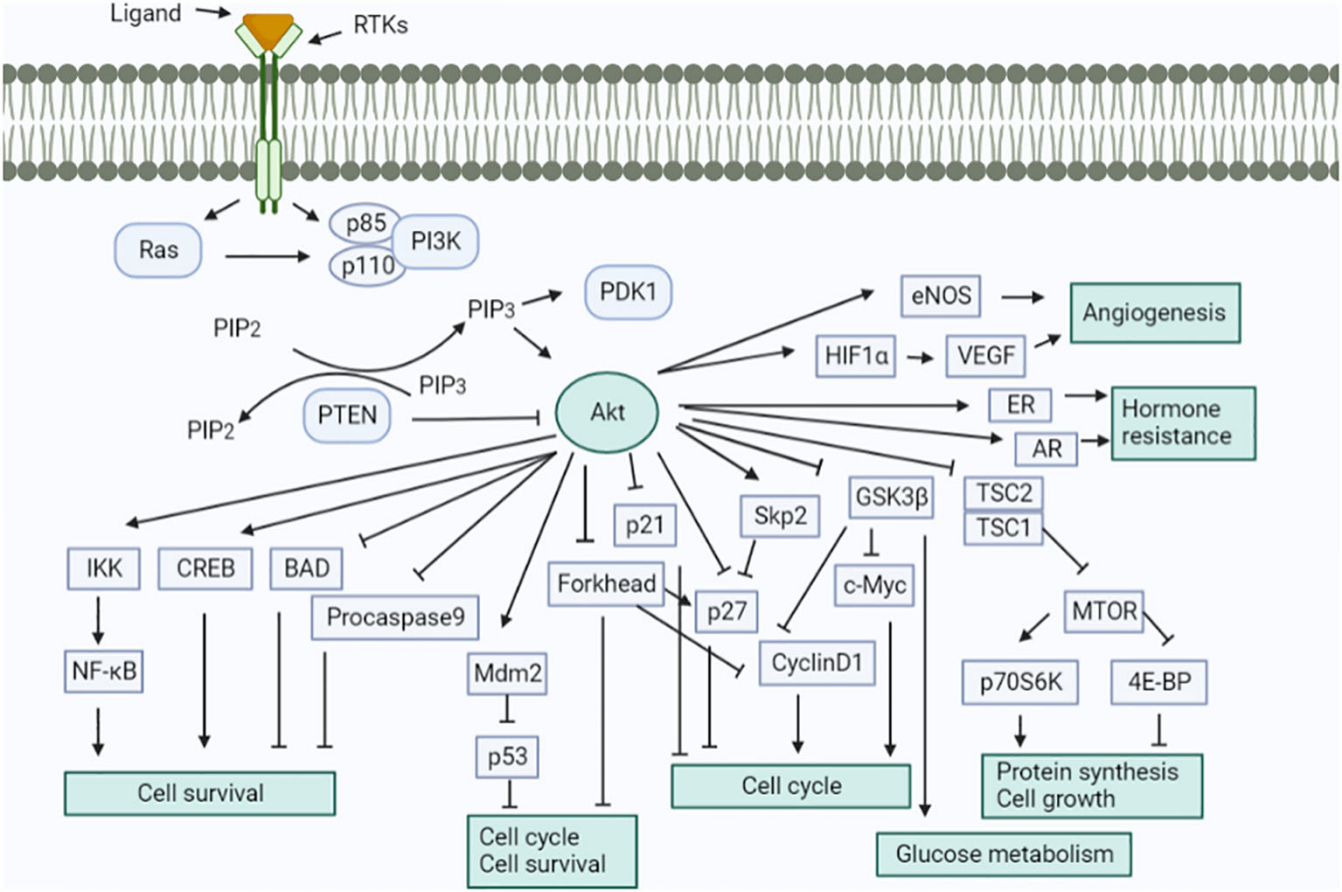
Schematic signaling shows the involvement of the renin–angiotensin system. Activation of the Akt signaling pathway in a variety of ways. This pathway performs numerous cellular tasks.

The important molecular pathways, such as the PI3K/PTEN/AKT/mTOR and RAS/RAF/MEK signaling pathways, have been examined for their participation in the development of endometrial cancer (**Figure 10**) (65, 66). Ras mutations can activate PI3K. The PI3K–AKT pathway is one of the most dysregulated signaling pathways in endometrial cancer, which is caused by mutations in tumor suppressor genes, i.e., PTEN and PIK3CA (67). These pathways are triggered by a variety of cytokines and growth factors to avoid apoptosis and cell proliferation. Numerous tumor types exhibit the abnormal regulation of PIP3K and RAS pathways brought on by mutations in Ras and B-Raf as well as other genes (such as PTEN, Akt, and PI3K). Therefore, different parts of these pathways are considered potential biological targets for cancer treatment.

### 8.1 Deregulation of signaling components

**Figure 11:**
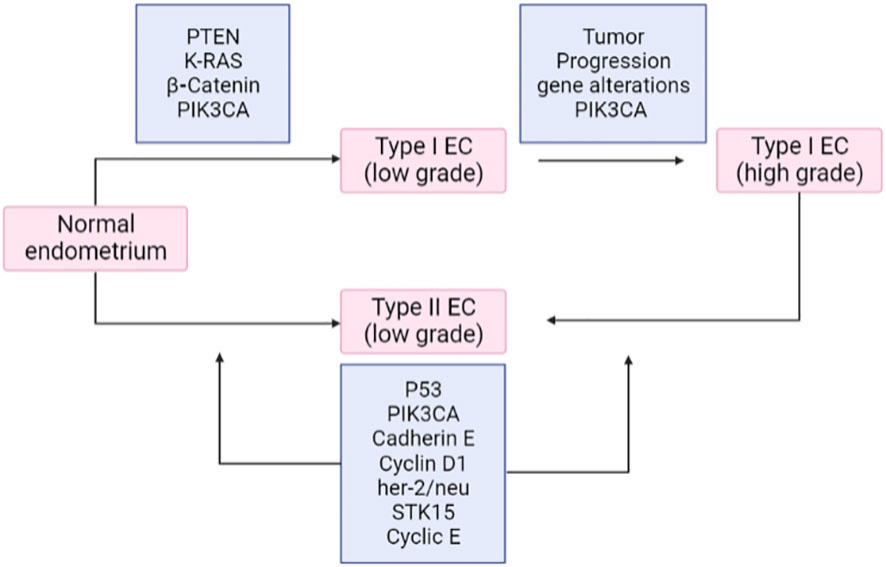
Frequent mutations in endometrial carcinoma. EC, endometrial cancer.

Deregulation of PI3K/PTEN/Akt/mTOR and RAS/RAF/MEK/ERK signaling cascades is frequently caused by epigenetic silencing or mutations in either upstream signaling molecules like receptor tyrosine kinases (RTKs) such as HER2, EGFR, IGF-1R, PDGFR, VEGF, FGFR2/3 or, in other components of the pathway, such as RAS, BRAF, NF1, MEK1, PIK3CA, PIK3 (R1, R4, and R5), mTOR, PTEN, Akt, IRS4, TSC1, and TSC2, as presented in **Figure 11**. It was previously considered that the ERK and MEK genes were seldom altered in human cancer, but according to recent research, MEK and MEK2 have been seen to be mutated in specific malignancies like in the case of ovarian and lung cancers. Deregulation of these components significantly impacts the differentiation pathways (68). It has been established that type I and type II endometrial cancers develop from different molecular alterations. Type I endometrial carcinoma shows mutations in K-RAS, PTEN, PIK3CA, and CTNNB1 (β-catenin) genes, whereas type II endometrial cancer has p53 changes, loss of heterozygosity, and other molecular modifications (p16, STK15, c-erb-B2, and E-cadherin). The RAS–RAF–MEK–ERK signaling pathway is crucial for tumorigenesis. The prevalence of K-RAS mutation varies from 10%–30%. It is believed that Ras effectors like RASSF1A offer an inhibitory growth signal that must be deactivated during tumorigenesis and remain inactivated. The increased activity of the RAS–RAF–MEK–ERK signaling pathway due to RASSFIA inactivation is caused by promoter hypermethylation (69). According to studies, the fibroblast growth factor (FGF) signaling pathway is significant in endometrial cancer. Endometrial cancer frequently exhibits the inactivation of the protein (SPRY-2) involved in the negative regulation of FGFR. Somatic mutations in FGFR2 receptor tyrosine kinase have also been reported to be around 6%–12%, specifically in type -I endometrial cancer (70, 71). Studies have concluded that mutations in PTEN and FGFR2 frequently coexist, whereas FGFR2 and K-RAS mutations remain mutually exclusive. In contrast, TP53 mutations occur more prominently in type II endometrial cancer than in type I, which significantly reduced the expression of c-erb-B2 (HER-2) and E-cadherin. Furthermore, variations in the STK15 gene have been indicated. The deregulation of E-cadherin is prominent in endometrial cancer and is brought on by promoter hypermethylation or loss of heterozygosity (72). The Akt pathway is negatively regulated by PTEN and its loss of function (due to deletion, mutation, or promoter methylation) leads to a rise in PIP3 concentration. The increased PIP3K substrate further results in the upregulation of the components of the PIP3K pathway, including Akt and mTOR.

### 8.2 Targeted therapies

There are no authorized targeted therapies for endometrial carcinoma. The prediction of advanced endometrial cancer in patients is still a problem. Recent developments in molecular targeted medicines have shown their potential to increase the cancer long-term survival rates of patients when used in conjunction with the right biomarkers. In this review, the anticancer effects of various pathway inhibitors are being clarified by preclinical and clinical investigations, although studies are ongoing in elucidating their effectiveness. Thus, **Figure 11** represents a comprehensive approach to inhibitors via different pathways in endometrial cancer.

### 8.3 PI3K/Akt/mTOR pathway inhibitors

**Figure 12:**
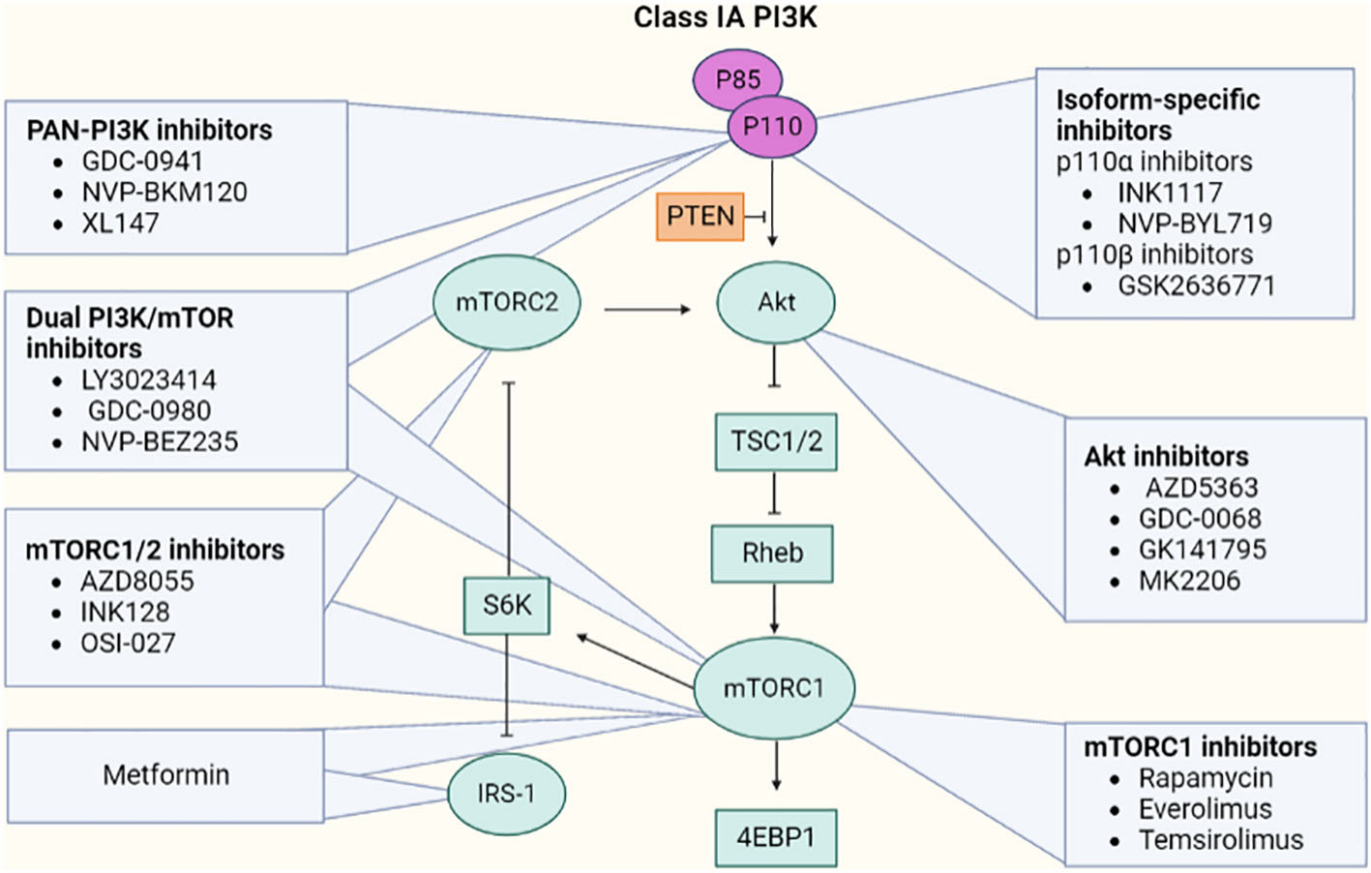
Representative inhibitors of the PI3k/Akt/mTOR pathway.

Many of the kinases found in the PI3K/Akt pathway provide excellent targets for the creation of small-molecule inhibitors. Additionally, multiple studies have demonstrated that compounds that block the PI3K/Akt pathway are likely to be utilized to treat not only endometrial carcinoma but also a variety of cancers (73). The inhibitors target the upstream regulators such as membrane receptors (like FGFR2) or directly block the steps of the pathway. The two varieties of PI3K inhibitor includes isoform-specific PI3K and Pan-PI3K inhibitors. The isoform-specific PI3K inhibitor such as buparlisib can inhibit all four isoforms of PI3K, whereas MLN1117 is a single specific isoform. Because of the major involvement of p110 (p110α and p110β) and p85 catalytic subunits, specific inhibitors that target them are considered for better safety assessment. The selective inhibitors for PI3K-p110α like INK1117 and NVP-BYL719 showed high efficacy in cell lines with PIK3CA mutations. The activity of PI3K-p110α inhibitors may be less effective against PTEN-deficient tumor cells. To overcome the reduced efficacy, GSK2636771 (p110β-specific inhibitor) or dual inhibitors of p110α and p110β could be employed [**Figure 12** (74)].

mTOR kinase may be found in two complexes—mTORC1 and mTORC2, and their inhibitors are also available. Rapamycin inhibits mTORC1 and may inhibit mTORC2 in specific cell types after extended incubation. Rapamycin has been shown to suppress angiogenesis and is an antiproliferative and anticancer agent. It reduces excess VEGF production. However, other analogs of rapamycin showed higher efficacy in cell lines with PIK3CA and/or PTEN mutations under the clinical trials of everolimus, temsirolimus, and ridaforolimus (75).

Akt has major relevance in activating mTORC1 by phosphorylation of TCS2 protein and inhibiting the AMP-PK enzyme that, in turn, activates Rheb and mTOR complex (75). A few of the Akt inhibitors include competitive inhibitors: AZD5363, GDC-0068, GSK2141795, MK2206 (allosteric inhibitor), miransertib (competes for the ATP binding site of AMP-PK), and perifosine that induces apoptosis.

Investigations revealed that second-generation mTOR inhibitors have a significant benefit. These dual mTOR inhibitors rather than targeting individual components can suppress the whole PI3K/Akt/mTOR pathway. Drugs such as vistusertib and sapanisertib concurrently inhibit the phosphorylation of Akt and S6K1. Some other dual inhibitors including LY3023414 and dactolisib can inhibit both mTOR complexes (mTORC1 and mTORC2) and all four catalytic isoforms of PI3K (76). The clinical trials of FGFR inhibitors revealed that it may not always have an oncogenic function; hence, its inhibition could lead to negative consequences. Furthermore, it has been investigated that the overexpression of a transmembrane protein EphA2 was seen in the majority of type I endometrial carcinoma. A microtubule inhibitor coupled with an anti-EphA2 monoclonal antibody, namely, MEDI-547, was employed in tumor-associated mice models, demonstrating a significant anticancer efficacy.

Moreover, the loss of function of PTEN in the PI3K pathway led to a hampered homologous recombination which makes the cells vulnerable to poly(ADP-ribose polymerase) (PARP) suppression. Research investigating PARP inhibitors is still ongoing (77). Regardless of the presence of other risk factors for this disease, type II diabetes shows a possible link with endometrial cancer (78). To treat such malignancies, metformin (an anti-diabetic drug) is being implied by the researchers. Metformin is supposed to control the PI3K/AKT/mTOR signaling through the activation of AMPK (79). Moreover, its therapeutic efficacy in treating different carcinomas is still under investigation.

## 9 Future perspective

Endometrial cancer, one of the most common gynecological malignancies in women representing 90% of uterine cancer, develops high chances of occurrence due to age, obesity, hypertension, and hyperestrogenism. This fundamentally deadliest cancer shows great relevance with the RAS which governs and maintains blood pressure, salt, water, and aldosterone secretion, thereby playing a significant role in the etiology of hypertension. The components such as ACE-I, ACE-II, AT1R, AT2R, and Pro(renin) are predominant components involved in RAS whose dysregulation in expression can lead to endometrial cancer. Thus, devising an efficient drug targeting the components of RAS could prove to be a promising scope to control the progression or development of endometrial cancer. Further studies are required to understand the mechanism behind the development of endometrial cancer through the dysregulation of RAS, and effective drugs need to be discovered to target the RAS system effectively. Additionally, another challenge faced in limiting the progression of cancer is its early diagnosis. Thus, certain predictive biomarkers with high specificity and sensitivity are required for early diagnosis of endometrial cancer. Moreover, the new era of artificial intelligence could also be a promising approach, changing the future of science in various public health sector diseases worldwide including cancer. Hence, devising effective AI tools for the diagnosis of cell progression, cell migration, and tumors in the region of endometrium can help to mitigate progressive endometrial cancer in women at the early stage.

## 10 Conclusion

A crosstalk between the pathways involved in endometrial cancer and the renin–angiotensin system shows the great relevance of different components of RAS in the progression and development of endometrial cancer in women above the age of 60. The presence of Pro(renin) in the ovarian follicular fluid makes it an effective target of choice to understand the various other pathways involved in the development of endometrial cancer through the dysregulation of RAS. Significantly, different pathways studied such as PI3K, TGF-β signaling, TNF signaling pathway, and ACEs are present to act as objectives of inhibitors and initiate endometrial cancer growth. The attributes of cancer such as tumor migration, invasion, and angiogenesis along with tumor adhesion to vascular endothelial cells are believed to be facilitated by angiotensin II. Various sets of genes involved in the pathogenesis of endometriosis include the AGTR1 gene. Thus, AT1R (receptor) plays a prominent role in the development and progression of endometrial cancer. Thereby, selective AT1 receptor blockers and ACE inhibitors (ACEIs) would target the RAS, providing therapeutic ramifications. Additionally, endometrial cancer is marked by the higher concentration of AT1R, ACE1, and ACE2 mRNA as compared to the surrounding non-cancerous tissues. ACE1 acts as a key enzyme in controlling the cell growth, division, and elimination of damaged cells; therefore, its dysregulation leads to uncontrolled growth and tumorigenesis. Prominently, ACE2 plays an important role in facilitating vasoconstriction and maintaining hydro-salinity balance and also in controlling follicular growth and ovulation. Moreover, Pro(renin) plays a versatile role in several malignancies, including endometrial cancer and therefore gets overexpressed in human endometrial cancer tissue, thereby stimulating angiogenesis and several other cellular processes such as proliferation and migration. The effectiveness and efficiency of RAS promote angiogenesis, cell proliferation, and migration in the endometrium under healthy conditions, especially during the menstrual phases, which concludes a positive relevance with the progression and development of endometrial cancer in women.

## Author contributions

NK, GR, DE, and ST played a role in designing the study as well as drafted the review paper. NK, AC, AF, AK, NS, and MK did the writing part. All authors contributed to the article and approved the submitted version.
